# Furthering the Focus on Translational Sleep Science in Behavioral Medicine

**DOI:** 10.1007/s12529-021-09979-9

**Published:** 2021-03-25

**Authors:** Michael A. Hoyt

**Affiliations:** 1 Department of Population Health & Disease Prevention, University of California, 653 E Peltason Drive, Irvine, CA 95697-3957 USA; 2 Chao Family Comprehensive Cancer Center, University of California, Irvine, USA; 3 Institute for Interdisciplinary Salivary Bioscience Research, University of California, Irvine, USA

**Keywords:** Sleep, Behavioral medicine

## Abstract

Translational sleep science has become a critical and fundamental focus in the field of behavioral medicine. This is the second issue in the special series of the *International Journal of Behavioral Medicine* focused on the physiological, psychological, social, and environmental concomitants of sleep and human health. The articles included in this issue draw further attention to the range and significance of sleep as a marker of health status and as a target of behavioral intervention. The research included in this series highlights the pervasive manner in which sleep health is intrinsically connected to health risk, behavior, and outcomes. The next decade promises to further behavioral medicine approaches to improving the provision of care and the overall public health through the implementation of translational sleep science research.

## Introduction

Empirical, theoretical, and clinical focus on translational sleep science in behavioral medicine continues to expand. This second special issue focused on sleep science in the *International Journal of Behavioral Medicine* is a testament to the growth in behavioral sleep research. The impact of sleep on human health is more apparent than ever before. The entire global community has experienced the enormous burden of the COVID-19 pandemic. Adjustment to prolonged changes and limitations in everyday life has impacted individuals' professional, social, emotional, physical, spiritual, and financial well-being [1]. Such behavioral changes, significant stressors, and challenged coping processes have profoundly altered the patterns and quality of our sleep and have brought acute awareness of the overall influence of sleep on our quality of life. A research focus on sleep health is both timely and paramount.

It has been well documented that much of the world experiences deficits in sleep [2] and that sleep problems provide multiple pathways to the development and/or exacerbation of myriad mental and physical health maladies [3]. The field of behavioral medicine has been rising to the challenge of recognizing and defining the health impact of sleep disruption and developing effective behavioral approaches to improving sleep patterns and quality. Research included in this special issue reflects strong examples of work that highlights the continued discovery of the bidirectional effects of sleep on health behaviors and health-relevant outcomes.

A critical task in translating basic behavioral research to effective interventions and clinical treatments involves identifying the groups and populations at heightened risk for experiencing the deleterious impact of poor sleep, as well as the individual and systemic predictors and precursors that perpetuate sleep problems. Many of the articles in this issue reveal the impact of sleep within specific groups. There is a strong emphasis on examining sleep in student groups and young adults. Jensen and colleagues ([4]; this issue) document associations of sleep quality and healthy eating behaviors in a relatively large sample of young adults. Likewise, Nicholson and colleagues ([5]; this issue) similarly connect poorer sleep quality with higher body mass index in college students. In fact, college students may be particularly vulnerable to poor sleep with impact on academic outcomes, physical health, and risk behavior. In this issue, Hamilton K. and colleagues ([6]; this issue) use a theoretically grounded approach to examine predictors of college student sleep across two cultural contexts and Hamilton N. and colleagues ([7]; this issue) use diary methods to identify the complex, reciprocal relationships of sleep and academic performance. The paper by Komrij and colleagues ([8]; this issue) highlights the contextual and behavioral predictors of sleep in one of the first large longitudinal cohort studies of child sleep duration.

The present issue includes a set of articles that highlight cross-cutting issues, research questions, and methods in translational sleep science. Included articles illustrate work that spans major research areas theories and clinical applications. Papers in this issue span across a variety of important groups at risk for poor sleep or vulnerability to the effects of sleep disruptions, including pregnant women ([9]; this issue), military personnel ([10]; this issue), as well as identification of individuals at heightened risk for cancer ([11]; this issue), and disordered weight control ([12]; this issue), or with multiple medical co-morbidities ([13]; this issue).

This special series of IJBM highlights the significance and innovation of sleep research in behavioral medicine as well as the opportunities for future work and the public health importance of sleep to our collective health. The next decade will likely continue to grow and develop translational sleep science, and behavioral medicine will focus on filling critical gaps in sleep knowledge and clinical practice. With developments in this arena so will come comprehensive improvements in clinical treatment of sleep disorders, increased knowledge of the risk factors for and biobehavioral mechanisms of sleep disruption, innovation in measurement and research design, optimization in impact and delivery of intervention, and mitigation of related health epidemics (e.g., obesity, cardiovascular disease) that are affected by sleep health. Sleep well.
